# Total Synthesis and Absolute Configuration of the Marine Norditerpenoid Xestenone

**DOI:** 10.3390/md7040654

**Published:** 2009-11-24

**Authors:** Koichiro Ota, Takao Kurokawa, Etsuko Kawashima, Hiroaki Miyaoka

**Affiliations:** School of Pharmacy, Tokyo University of Pharmacy and Life Sciences, 1432-1 Horinouchi, Hachioji, Tokyo 192-0392, Japan; E-Mails: otak@toyaku.ac.jp (K.O.); kawasima@toyaku.ac.jp (E.K.)

**Keywords:** xestenone, marine norditerpene, total synthesis, structural determination

## Abstract

Xestenone is a marine norditerpenoid found in the northeastern Pacific sponge *Xestospongia vanilla*. The relative configuration of C-3 and C-7 in xestenone was determined by NOESY spectral analysis. However the relative configuration of C-12 and the absolute configuration of this compound were not determined. The authors have now achieved the total synthesis of xestenone using their developed one-pot synthesis of cyclopentane derivatives employing allyl phenyl sulfone and an epoxy iodide as a key step. The relative and absolute configurations of xestenone were thus successfully determined by this synthesis.

## 1. Introduction

**Figure 1.**
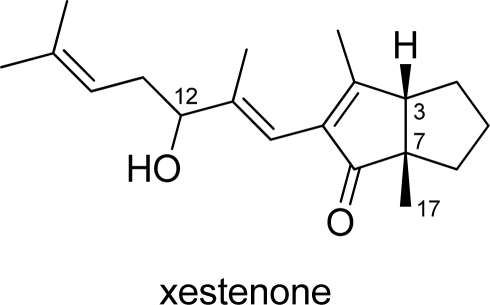
Structure of xestenone.

The norditerpenoid xestenone (Figure 1) was first isolated from the marine sponge *Xestospongia vanilla* in 1988 [1]. Its planar structure was determined by ^1^H- and ^13^C-NMR and mass spectral analysis. The stereochemistry comprises two *cis* fused cyclopentane rings, as determined by the NOE correlation between the methyl protons at C-17 and the methine proton at C-3, although the relative configuration of C-12 and the absolute configuration were not determined. Moreover, no biological activity has been reported for xestenone, although various bioactive compounds that have been isolated from several *Xestospongia* sponges [2].

**Scheme 1.**
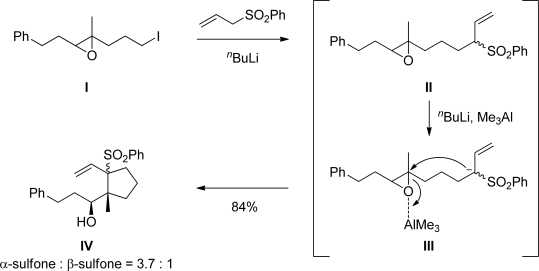
One-pot synthesis of cyclopentane derivatives.

The authors recently reported a stereocontrolled one-pot synthesis of cyclopentane derivatives possessing a quaternary carbon, which involved: 1) reaction of an anion derived from allyl phenyl sulfone with epoxy iodide **I** to give epoxysulfone **II**; 2) *in situ* deprotonation of **II** to generate an epoxysulfone anion **III** and 3) intramolecular cyclization to give cyclopentane derivative **IV** (Scheme 1) [3]. This one-pot synthesis of cyclopentane derivatives has now been applied to the total synthesis of xestenone, and in this paper the authors wish to report on the successful total synthesis of xestenone and its complete structural determination.

## 2. Results and Discussion

### 2.1. Retrosynthetic analysis

**Scheme 2.**
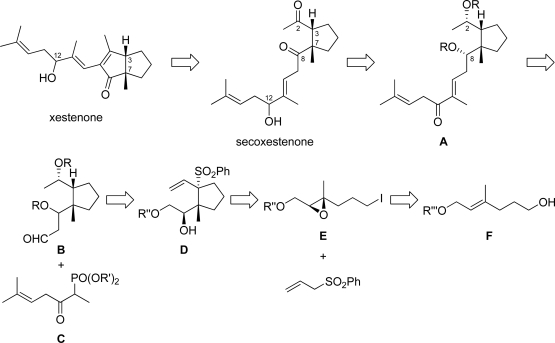
Retrosynthetic analysis of xestenone.

The authors planned to synthesize both xestenone and 12-*epi*-xestenone, since the relative configuration at C-12 of xestenone was unknown at the onset (Scheme 2). Xestenone was obtained from secoxestenone by intramolecular aldol condensation [4]. Secoxestenone would be obtained from α,β-unsaturated ketone **A** by 1,2-reduction of α,β-unsaturated ketone at C-12 and oxidation of the hydroxy group at C-2 and C-8. The α,β-unsaturated ketone **A** would be obtained from aldehyde **B** by the Horner-Wadsworth-Emmons reaction using known phosphonate **C** [5]. For the right hand fragment of xestenone, aldehyde **B** would be synthesized from cyclopentane **D** through various chemical functionalizations. Cyclopentane **D** would be constructed by our developed stereocontrolled one-pot synthesis of cyclopentane derivatives using trisubstituted epoxy iodide **E** and allyl phenyl sulfone [3]. Epoxy iodide **E** would be obtained from alcohol **F**.

### 2.2. Synthesis of xestenone

**Scheme 3.**
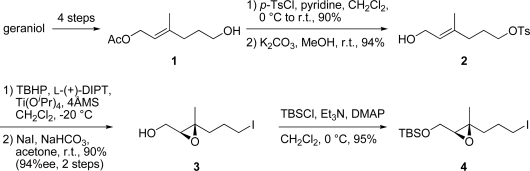
Synthesis of epoxy iodide **4**.

Geraniol was converted to alcohol **1** by known procedures [6]. Alcohol **1** was treated with *p*-TsCl and pyridine to give the corresponding tosylate (90%), which was deacetylated with K_2_CO_3_ in MeOH to furnish allylic alcohol **2** (94%; Scheme 3). Allylic alcohol **2** was converted to chiral β-epoxyalcohol using Sharpless asymmetric epoxidation under standard conditions [7] (94% ee). Iodination of the epoxy alcohol with NaI and NaHCO_3_ furnished epoxy iodide **3** (90%, 2 steps). Protection of the primary hydroxy group in **3** as the TBS ether gave the desired chiral epoxy iodide **4** (95%).

**Scheme 4.**
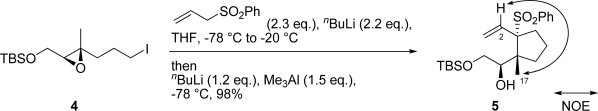
Synthesis of intermediate **5**.

The sulfonyl carbanion prepared from allyl phenyl sulfone (2.3 eq.) and *^n^*BuLi (2.2 eq.) was reacted with epoxy iodide **4** at −20 °C. Following confirmation of the disappearance of **4** by TLC, *^n^*BuLi (1.2 eq.) and Me_3_Al (1.5 eq.) were added at −78 °C to give cyclopentane **5** as the sole product (98%; Scheme 4). The *trans*-configuration of the vinyl and 1-hydroxy-2-silyloxyethyl groups in cyclopentane **5** was determined by NOE correlation between the vinyl proton at C-2 and methyl protons at C-17. The stereoselectivity of this reaction is presumably the result of steric hindrance between the phenyl sulfonyl group and the 1-hydroxy-2-silyloxyethyl group in the intermediate sulfonyl carbanion.

**Scheme 5.**
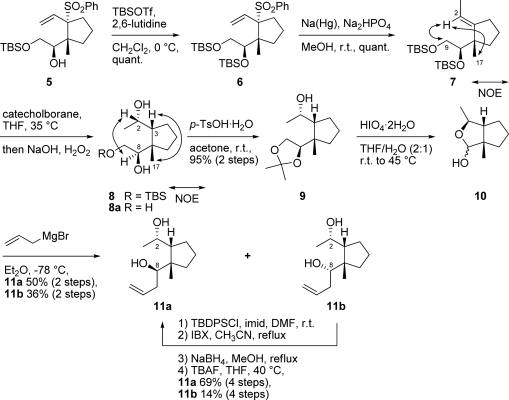
Synthesis of diols **11a** and **11b**.

**Scheme 6.**
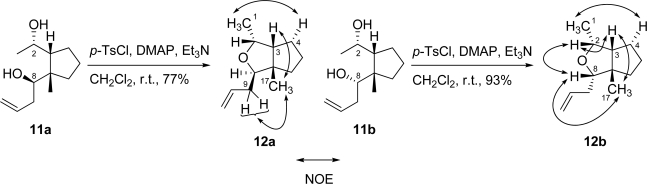
Determination of the relative configuration of **11a** and **11b**.

Protection of the secondary hydroxy group of **5** with TBSOTf and 2,6-lutidine (Scheme 5) furnished bis-silyl ether **6** (quant.). The phenylsulfonyl group in **6** was removed by treatment with Na(Hg) and Na_2_HPO_4_ to give trisubstituted (*E*)-olefin **7** as a sole product (quant.). The *E*-configuration of the trisubstituted olefin in **7** was determined by NOE correlation between the vinyl proton at C-2 and methyl protons at C-17, and the vinyl proton at C-2 and methylene protons at C-9. Diastereoselective hydroboration-oxidation of *E*-olefin **7** with catecholborane furnished a mixture of diol **8** and triol **8a**. The stereochemistry of diol **8** was elucidated by NOESY spectral analysis. The NOE correlation between the methyl protons at C-17 and methine proton at C-3, and the methine proton at C-2 and methine proton at C-8 in diol **8** suggested that the methyl group and methine proton of cyclopentane were oriented in the same β-configuration. The mixture of diol **8** and triol **8a** was treated with *p*-TsOH·H_2_O in acetone to give acetonide **9** (95%, 2 steps). Subsequent deprotection of the acetonide group in **9** and oxidative cleavage of the 1,2-diol with HIO_4_·2H_2_O afforded hemiacetal **10**, which was converted to homoallylic alcohols **11a** (50%, 2 steps) and **11b** (36%, 2 steps). These alcohols were easily separated by silica gel chromatography. The relative configurations of these homoallylic alcohols **11a** and **11b** were determined by chemical conversion and NOESY spectral analysis. Compounds **11a** and **11b** were converted to tetrahydrofurans **12a** and **12b** by treatment with *p*-TsCl, DMAP and Et_3_N (Scheme 6). The NOE correlations of **12a** between the methylene protons at C-9 and methyl protons at C-17, and one of the methylene protons at C-4 and methyl protons at C-1 suggested that the C-1 methyl group and allyl group were oriented on different faces of the tetrahydrofuran ring, therefore, the stereochemistry of the hydroxy group at C-8 in homoallylic alcohol **11a** was found to adopt a β-configuration. The NOE correlations of **12b** between the methine proton at C-2 and methine proton at C-3, the methine proton at C-2 and methine proton at C-8, the methine proton at C-8 and methyl protons at C-17, the methine proton at C-3 and methyl protons at C-17, and the methyl protons at C-1 and one of the methylene protons at C-4 were observed. The results suggested that the C-1 methyl group and allyl group were oriented on the same face of the tetrahydrofuran ring. Therefore, the stereochemistry of the hydroxy group at C-8 in homoallylic alcohol **11b** was found to adopt an α-configuration. Both homoallylic alcohols **11a** and **11b** could be converted to xestenone. However, the chemical yield of the later steps in this synthesis from α-alcohol **11b** was low. Therefore, α-alcohol **11b** was converted into β-alcohol **11a** by inversion of the C-8 stereocenter. The hydroxy group at C-2 in α-alcohol **11b** was protected with TBDPSCl and imidazole to give TBDPS ether, which was oxidized with IBX [8] to afford the ketone. Diastereoselective reduction of the ketone with NaBH_4_ to the alcohol, followed by deprotection of the TBDPS group with TBAF furnished a mixture of the desired β-alcohol **11a** (69%, 4 steps) and α-alcohol **11b** (14%, 4 steps).

**Scheme 7.**
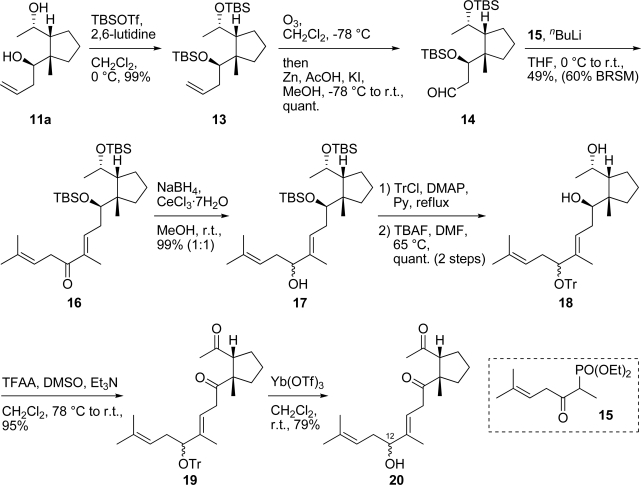
Synthesis of diketone **20**.

β-Alcohol **11a** was converted to bis-silyl ether **13** by treatment with TBSOTf and 2,6-lutidine (99%), and was followed by ozonolysis to give aldehyde **14** (quant.), thereby completing the synthesis of the right hand fragment of xestenone in 14 steps from known alcohol **1** [6] (Scheme 7). Treatment of aldehyde **14** with the anion of phosphonate **15** [5] in THF at r.t. provided α,β-unsaturated ketone **16** (49%). 1,2-Reduction of the α,β-unsaturated ketone **16** with NaBH_4_ in the presence of CeCl_3_·7H_2_O in MeOH [9] furnished allylic alcohol **17** as an inseparable mixture (99%, 1:1). Protection of the hydroxy group in allylic alcohol **17** with TrCl and DMAP in pyridine provided the trityl ether, and was followed by desilylation with TBAF to give diol **18** (quant., 2 steps). Oxidation of two hydroxy groups in diol **18** with TFAA, DMSO and Et_3_N generated diketone **19** (95%). Lewis acid-mediated deprotection of the trityl group in diketone **19** with Yb(OTf)_3_ furnished diketone **20** as an inseparable diastereomeric mixture of the hydroxy group at C-12 (79%). Diketone **20** corresponds to secoxestenone.

**Scheme 8.**
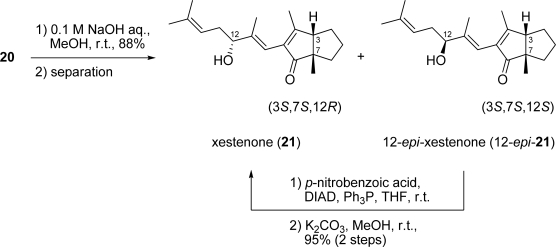
Synthesis of xestenone (**21**).

Finally, intramolecular aldol condensation of diketone **20** was achieved by treatment with 0.1 M NaOH aq. to furnish a diastereomeric mixture of xestenone **21** and 12-*epi*-**21** (88%; Scheme 8). Separation of the diastereomeric mixture using a chiral HPLC column gave **21** {[α]_D_^25^ +2.2° (*c* 0.08, MeOH)}, and 12-*epi*-**21** {[α]_D_^25^ −113.7° (*c* 0.09, MeOH)}. The optical rotation of synthetic **21** is not identical, but very close to the value obtained for the natural product {[α]_D_ 0° (*c* 1.00, MeOH)} [1]. Moreover, the CD spectrum of the synthetic **21** matched that of the natural product [1]. The CD spectrum of the synthetic **21** showed a positive Cotton effect at 323 nm and a negative Cotton effect at 258 nm. The absolute configuration of the hydroxy group at C-12 in **21** was determined by comparing the ^1^H-NMR data of the two diastereomeric esters (MPA esters) [10]. The absolute configuration of the hydroxy group at C-12 in 12-*epi***-21** was determined by the same method. As a result, the absolute stereochemistry of the three chiral centers in xestenone was determined to be 3*S*, 7*S* and 12*R*. 12-*epi*-xestenone (12-*epi*-**21**) was converted to xestenone (**21)** by a Mitsunobu reaction.

## 3. Experimental Section

### 3.1. General

Optical rotations were measured using a Jasco P-1030 polarimeter. Melting points (mp) were measured using a Yazawa melting point apparatus BY-2 and are uncorrected. IR spectra were recorded using a Jasco FT-IR/620 spectrometer. UV spectra were recorded using a Jasco V-550 spectrophotometer. Circular dichroism (CD) spectra were measured with a Jasco J-720 spectropolarimeter. ^1^H- and ^13^C-NMR spectra were recorded on a Bruker DRX-400 or a Bruker Biospin AV-600 spectrometer. Chemical shifts are given on the δ (ppm) scale using tetramethylsilane (TMS) as the internal standard (s, singlet; d, doublet; t, triplet; q, quartet; quint, quintet; m, multiplet; br, broad). High resolution ESIMS (HRESIMS) spectra were obtained using a Micromass LCT spectrometer. Elemental analysis data were obtained using an Elemental Vavio EL. Flash column chromatography was performed using Kanto Chemical Silica Gel 60N (spherical, neutral) 40–50 μm.

### 3.2. (E)-6-Hydroxy-4-methylhex-4-enyl 4-methylbenzenesulfonate (**2**)

To a solution of (*E*)-6-hydroxy-3-methylhex-2-enyl acetate [6] (**1**, 530 mg, 3.08 mmol) in CH_2_Cl_2_ (10.3 mL) were added pyridine (374 μL, 4.62 mmol) and *p*-toluenesulfonyl chloride (705 mg, 3.70 mmol) at 0 °C. After stirring for 5 hr at r.t., the mixture was diluted with Et_2_O, washed with H_2_O and brine, and then dried. Removal of the solvent gave a residue which was then purified by silica gel column chromatography (hexane/AcOEt = 2:1) to generate tosylate (905 mg, 90% yield) as a colorless oil. IR (neat) 2924, 1733, 1359 cm^−1^; ^1^H-NMR (400 MHz, CDCl_3_) δ ppm: 7.77 (2H, d, *J* = 8.2 Hz), 7.33 (2H, d, *J* = 8.2 Hz), 5.25 (1H, m), 4.52 (2H, d, *J* = 7.2 Hz), 4.01 (2H, t, *J* = 6.4 Hz), 2.44 (3H, s), 2.05 (2H, t, *J* = 7.2 Hz), 2.03 (3H, s), 1.77 (2H, m), 1.64 (3H, s); ^13^C-NMR (100 MHz, CDCl_3_) δ ppm: 170.9, 144.7, 133.3, 129.8, 127.8, 119.6, 69.8, 61.0, 35.0, 26.8, 21.5, 20.9, 16.2; HRESIMS (*m*/*z*) calcd. for C_16_H_23_O_5_S (M+H)^+^ 349.1086, found 349.1086; Anal. Calcd. for C_16_H_22_O_5_S: C, 58.87; H, 6.79. Found: C, 58.94; H, 6.75.

To a solution of the above tosylate (9.47 g, 29.0 mmol) in MeOH (290 mL) was added K_2_CO_3_ (4.81 g, 34.8 mmol) at r.t. After stirring for 30 min at the same temperature, the mixture was diluted with Et_2_O and then filtered through a silica gel pad. Removal of the solvent gave a residue which was then purified by silica gel column chromatography (hexane/AcOEt = 2:1) to generate allylic alcohol **2** (7.74 g, 94% yield) as a colorless oil. IR (neat) 3387, 2923, 1354 cm^−1^; ^1^H-NMR (400 MHz, CDCl_3_) δ ppm: 7.75 (2H, d, *J* = 8.2 Hz), 7.32 (2H, d, *J* = 8.2 Hz), 5.31 (1H, m), 4.07 (2H, d, *J* = 6.5 Hz), 4.00 (2H, t, *J* = 6.5 Hz), 2.42 (3H, s), 2.02 (2H, t, *J* = 7.8 Hz), 1.76 (2H, m), 1.59 (3H, s), 1.52 (1H, br s); ^13^C-NMR (100 MHz, CDCl_3_) δ ppm: 144.7, 137.2, 133.2, 129.8, 127.8, 124.7, 69.8, 59.0, 35.0, 26.6, 21.5, 15.9; HRESIMS (*m*/*z*) calcd. for C_14_H_21_O_4_S (M+H)^+^ 307.0980, found 307.0994; Anal. Calcd. for C_14_H_20_O_4_S: C, 59.13; H, 7.09. Found: C, 59.07; H, 7.06.

### 3.3. ((2S,3S)-3-(3-Iodopropyl)-3-methyloxiran-2-yl)methanol (**3**)

To a cold (−20 °C) suspension of 4Å molecular sieves (114 mg) in CH_2_Cl_2_ (1.6 mL) were added L-(+)-DIPT (5.2 μL, 24.8 μmol), Ti(O*^i^*Pr)_4_ (6.2 μL, 21.0 μmol) and TBHP (164 μL, 101 mmol, 6.17 M in CH_2_Cl_2_ solution). After stirring for 30 min at the same temperature, a solution of allylic alcohol **2** (54.4 mg, 191 μmol) in CH_2_Cl_2_ (500 μL) was added over 5 min. After stirring at −20 °C for 15 min, NaOH (13.0 μL, 30% in saturated aqueous NaCl) was added. The mixture was diluted with Et_2_O, warmed to r.t. and stirred for 10 min. MgSO_4_ (11.6 mg) and Celite (1.4 mg) were then added and after stirring for 15 min, the mixture was filtered through a Celite pad and the filtrate was concentrated under reduced pressure to afford the crude epoxide. To a solution of the crude epoxide in acetone (1.9 mL) were added NaHCO_3_ (17.7 mg, 210 μmol) and NaI (286 mg, 1.91 mmol) at r.t. After stirring for 8 hr at the same temperature, the mixture was diluted with Et_2_O and then filtered through a silica gel pad. Removal of the solvent gave a residue which was then purified by silica gel column chromatography (hexane/AcOEt = 1:2) to generate epoxy iodide **3** (44.0 mg, 90% yield) as a yellow oil. [α]_D_^28^ −10.0 (*c* 1.03, CHCl_3_); IR (neat) 3418, 2929 cm^−1^; ^1^H-NMR (400 MHz, CDCl_3_) δ ppm: 3.79 (1H, m), 3.68 (1H, m), 3.18 (2H, m), 2.97 (2H, t, *J* = 5.4 Hz), 1.99 (1H, br s), 1.93 (2H, m), 1.64 (2H, m), 1.29 (3H, s); ^13^C-NMR (100 MHz, CDCl_3_) δ ppm: 62.6, 61.2, 60.3, 39.0, 29.0, 16.8, 5.8; HRESIMS (*m*/*z*) calcd. for C_7_H_12_IO (M-OH)^+^ 238.9933, found 238.9930; Anal. Calcd. for C_7_H_13_IO_2_: C, 32.83; H, 5.12. Found: C, 33.06; H, 5.26.

### 3.4. tert-Butyl(((2S,3S)-3-(3-iodopropyl)-3-methyloxiran-2-yl)methoxy)dimethylsilane (**4**)

To a solution of epoxy iodide **3** (387 mg, 1.51 mmol) in CH_2_Cl_2_ (1.5 mL) were added Et_3_N (253 mg, 1.82 mmol), DMAP (185 mg, 1.51 mmol) and TBSCl (251 mg, 1.82 mmol) and the mixture was stirred at r.t. for 30 min. The mixture was diluted with Et_2_O, washed with saturated aqueous NaHCO_3_ solution, H_2_O and brine, and then dried. Removal of the solvent gave a residue which was then purified by silica gel column chromatography (hexane/AcOEt = 7:1) to generate epoxy iodide **4** (530 mg, 95% yield) as a colorless oil. [α]_D_^25^ +6.9 (*c* 1.06, CHCl_3_); IR (neat) 2929 cm^−1^; ^1^H-NMR (400 MHz, CDCl_3_) δ ppm: 3.76 (1H, dd, *J* = 11.5, 5.5 Hz), 3.69 (1H, dd, *J* = 11.5, 5.5 Hz), 3.19 (2H, t, *J* = 7.0 Hz), 2.91 (1H, t, *J* = 5.5 Hz), 1.95 (2H, m), 1.64 (2H, m), 1.26 (3H, s), 0.91 (9H, s), 0.07 (6H, s); ^13^C-NMR (100 MHz, CDCl_3_) δ ppm: 62.8, 62.0, 59.5, 39.0, 29.2, 25.9, 18.3, 16.7, 5.8, −5.2, −5.4; HRESIMS (*m*/*z*) calcd. for C_13_H_28_IO_2_Si (M+H)^+^ 371.0903, found 371.0921; Anal. Calcd. for C_13_H_27_IO_2_Si: C, 42.16; H, 7.35. Found: C, 42.37; H, 7.23.

### 3.5. (R)-1-[(1R,2S)-2-Benzenesulfonyl-1-methyl-2-vinylcyclopentyl]-2-(tert-butyldimethylsiloxy) ethanol (**5**)

To a solution of allyl phenyl sulfone (95.2 mg, 0.552 mmol) in THF (3.0 mL) was added *^n^*BuLi (317 μL, 0.500 mmol, 1.58 M in hexane solution) at −78 °C and the mixture was warmed to 0 °C. The mixture was stirred for 30 min at the same temperature. After cooling to −78 °C, a solution of epoxy iodide **4** (84.1 mg, 0.227 mmol) in THF (1.6 mL) was added and the mixture was warmed to −20 °C. The mixture was stirred for 30 min at the same temperature. After cooling to −78 °C, *^n^*BuLi (173 μL, 0.273 mmol, 1.58 M in hexane solution) was added and the mixture was warmed to −20 °C. The mixture was stirred for 30 min at the same temperature. After cooling to −78 °C, Me_3_Al (331 μL, 0.341 mmol, 1.03 M in hexane) was added. After stirring for 1 hr at the same temperature, the mixture was diluted with Et_2_O, washed with saturated aqueous NH_4_Cl solution, H_2_O and brine, and then dried. Removal of the solvent gave a residue which was then purified by silica gel column chromatography (hexane/AcOEt = 10:1) to generate cyclopentane **5** (94.4 mg, 98% yield) as a white solid. mp 125–126 °C; [α]_D_^25^ −114 (*c* 0.81, CHCl_3_); IR (KBr) 3560, 2952 cm^−1^; ^1^H-NMR (400 MHz, CDCl_3_) δ ppm: 7.78 (2H, m), 7.57 (1H, m), 7.45 (2H, m), 6.19 (1H, dd, *J* = 17.4, 10.9 Hz), 5.25 (1H, d, *J* = 10.9 Hz), 4.73 (1H, d, *J* = 17.4 Hz), 4.56 (1H, dt, *J* = 6.2, 2.9 Hz), 4.29 (1H, dd, *J* = 9.5, 3.5 Hz), 3.67 (1H, t, *J* = 8.8 Hz), 3.23 (1H, d, *J* = 2.9 Hz), 2.57 (1H, m), 2.06 (3H, m), 1.73 (2H, m), 0.99 (3H, s), 0.93 (9H, s), 0.14 (6H, s); ^13^C-NMR (100 MHz, CDCl_3_) δ ppm: 137.4, 135.3, 133.3, 130.6, 127.9, 120.3, 81.0, 74.3, 64.2, 54.6, 37.2, 30.8, 25.9, 20.0, 19.6, 18.2, −5.1, −5.3; HRESIMS (*m*/*z*) calcd. for C_22_H_37_O_4_SSi (M+H)^+^ 425.2182, found 425.2179; Anal. Calcd. for C_22_H_36_O_4_SSi: C, 62.22; H, 8.54. Found: C, 62.11; H, 8.41.

### 3.6. {(1S,2R)-2-[(R)-1,2-Bis(tert-butyldimethylsiloxy)ethyl]-2-methyl-1-vinylcyclopentanesulfonyl} benzene (**6**)

To a solution of cyclopentane **5** (7.01 g, 16.5 mmol) in CH_2_Cl_2_ (16.5 mL) were added 2,6-lutidine (17.7 g, 165 mmol) and TBSOTf (7.02 g, 26.6 mmol) and the mixture was stirred at 0 °C for 30 min. The mixture was diluted with Et_2_O, washed with saturated aqueous NaHCO_3_ solution, H_2_O and brine, and then dried. Removal of the solvent gave a residue which was then purified by silica gel column chromatography (hexane/AcOEt = 5:1) to generate bis-silyl ether **6** (8.89 g, quantitative yield) as a colorless oil. [α]_D_^25^ −77.9 (*c* 1.62, CHCl_3_); IR (neat) 2954, 1133 cm^−1^; ^1^H-NMR (400 MHz, CDCl_3_) δ ppm: 7.78 (2H, m), 7.57 (1H, m), 7.45 (2H, m), 6.37 (1H, dd, *J* = 17.4, 10.9 Hz), 5.27 (1H, d, *J* = 10.9), 4.72 (1H, d, *J* = 17.4 Hz), 4.63 (1H, dd, *J* = 5.9, 1.8 Hz), 4.15 (1H, dd, *J* = 10.5, 1.8 Hz), 3.87 (1H, dd, *J* = 10.5, 5.9), 2.51 (1H, m), 2.12 (1H, m), 2.01 (1H, m), 1.90 (3H, m), 0.92 (21H, m), 0.16 (12H, m); ^13^C-NMR (100 MHz, CDCl_3_) δ ppm: 137.3, 135.3, 133.2, 130.7, 127.9, 120.7, 81.1, 77.2, 66.5, 56.3, 40.0, 31.6, 26.3, 26.2, 19.8, 19.0, 18.5, −3.3, −4.6, −5.1, −5.4; HRESIMS (*m*/*z*) calcd. for C_28_H_51_O_4_SSi_2_ (M+H)^+^ 539.3047, found 539.3086; Anal. Calcd. for C_28_H_50_O_4_SSi_2_: C, 62.40; H, 9.35. Found: C, 62.37; H, 9.11.

### 3.7. (S)-1-[(R)-1,2-Bis(tert-butyldimethylsiloxy)ethyl]-2-ethylidene-1-methylcyclopentane (**7**)

To a solution of bis-silyl ether **6** (9.29 g, 17.2 mmol) in MeOH (344 mL) were added Na_2_HPO_4_ (17.1 g, 120.5 mmol) and 5% Na(Hg) (31.6 g). After stirring for 1 hr at r.t., the mixture was diluted with Et_2_O and filtered through silica gel. The filtrate was then concentrated under reduced pressure. The resultant residue was then purified by silica gel column chromatography (hexane only) to generate *E*-olefin **7** (6.86 g, quantitative yield) as a colorless oil. [α]_D_^25^ +19.0 (*c* 1.35, CHCl_3_); IR (neat) 2955 cm^−1^; ^1^H-NMR (400 MHz, CDCl_3_) δ ppm: 5.17 (1H, m), 3.78 (1H, dd, *J* = 10.3, 2.8 Hz), 3.52 (1H, dd, *J* = 9.2, 1.8 Hz), 3.46 (1H, dd, *J* = 10.3, 5.8 Hz), 2.35 (1H, m), 2.08 (2H, m), 1.67 (1H, m), 1.58 (3H, d, *J* = 6.7 Hz), 1.53 (2H, m), 1.22 (1H, m), 0.97 (3H, s), 0.88 (9H, s), 0.86 (9H, s), 0.07 (3H, s), 0.03 (3H, s), 0.03 (3H, s), 0.00 (3H, s); ^13^C-NMR (100 MHz, CDCl_3_) δ ppm: 150.2, 114.1, 79.1, 66.3, 49.5, 35.7, 30.0, 26.1, 26.0, 23.9, 22.5, 18.4, 18.3, 14.7, −3.9, −5.0, −5.3; HRESIMS (*m*/*z*) calcd. for C_22_H_46_O_2_Si_2_Na (M+Na)^+^ 421.2934, found 421.2914; Anal. Calcd. for C_22_H_46_O_2_Si_2_: C, 66.26; H, 11.63. Found: C, 66.36; H, 11.50.

### 3.8. (S)-1-{(1R,2S)-2-[(R)-2,2-Dimethyl-[1,3]dioxolan-4-yl]-2-methylcyclopentyl}ethanol (**9**)

To a solution of *E*-olefin **7** (3.86 g, 9.69 mmol) in THF (19.4 mL) was added catecholborane (6.40 mL, 60.1 mmol) dropwise at 0 °C. After stirring for 12 hr at the same temperature, 1M NaOH solution (12.9 mL) and 35% aqueous H_2_O_2_ solution (36.9 mL) were added to the mixture at r.t. After stirring for 2 hr, the resultant mixture was diluted with CHCl_3_, washed with H_2_O and brine, dried and then concentrated to afford a mixture of diol **8** and triol **8a**. To a solution of the crude alcohols in acetone (96.9 mL) was added *p*-TsOH·H_2_O (735 mg, 3.88 mmol) at r.t. After stirring for 2 hr at the same temperature, the mixture was diluted with Et_2_O, washed with saturated aqueous NaHCO_3_ solution, H_2_O and brine, and then dried. Removal of the solvent gave a residue which was then purified by silica gel column chromatography (hexane/acetone = 4:1) to generate acetonide **9** (2.11 g, 95% yield) as a colorless oil. [α]_D_^25^ +4.0 (*c* 0.58, CHCl_3_); IR (neat) 3443, 2956 cm^−1^; ^1^H-NMR (400 MHz, CDCl_3_) δ ppm: 4.42 (1H, dd, *J* = 8.5, 6.5 Hz), 4.01 (1H, m), 3.96 (1H, dd, *J* = 7.8, 6.5 Hz), 3.66 (1H, t, *J* = 8.2 Hz), 2.07 (1H, s), 1.89 (1H, m), 1.69–1.50 (4H, m), 1.41 (3H, s), 1.35 (3H, s), 1.37–1.33 (2H, m), 1.20 (3H, d, *J* = 6.2 Hz), 1.07 (3H, s); ^13^C-NMR (100 MHz, CDCl_3_) δ ppm: 108.5, 79.9, 69.3, 66.9, 59.1, 45.1, 35.0, 31.2, 27.8, 26.5, 25.3, 24.2, 23.3; HRESIMS (*m*/*z*) calcd. for C_13_H_25_O_3_ (M+H)+ 229.1804, found 229.1810; Anal. Calcd. for C_13_H_24_O_3_: C, 68.38; H, 10.59. Found: C, 68.43; H, 10.59.

### 3.9. (R)-1-[(1S,2R)-2-((S)-1-Hydroxyethyl)-1-methylcyclopentyl]but-3-en-1-ol (**11a**) and (S)-1-[(1S,2R)-2-((S)-1-hydroxyethyl)-1-methylcyclopentyl]but-3-en-1-ol (**11b**)

To a solution of acetonide **9** (2.11 g, 9.24 mmol) in THF was added a solution of HIO_4_2H_2_O (12.6 g, 55.4 mmol) in H_2_O (93.0 mL) at r.t. After stirring for 3 hr at 45 °C, the mixture was diluted with Et_2_O, washed with H_2_O and brine, dried and then concentrated to afford crude hemiacetal **10**. To a solution of crude hemiacetal **10** in Et_2_O (93.0 mL) was added allyl magnesium bromide (33.0 mL, 32.3 mmol, 1.0 M in Et_2_O solution) at −78 °C. After stirring for 1 hr at 0 °C, the mixture was diluted with Et_2_O, washed with saturated aqueous NH_4_Cl solution, H_2_O and brine, and then dried. Removal of the solvent gave a residue which was then purified by silica gel column chromatography (hexane/AcOEt = 8:1) to generate diol **11a** (914 mg, 50% yield) as a colorless oil and diol **11b** (650 mg, 36 % yield) as a white solid. Compound **11a**: [α]_D_^25^ −5.0 (*c* 0.39, CHCl_3_); IR (neat) 3306, 2955 cm^−1^; ^1^H-NMR (400 MHz, CDCl_3_) δ ppm: 5.87 (1H, m), 5.19 (1H, d, *J* = 10.4 Hz), 5.18 (1H, d, *J =* 16.8 Hz), 3.86 (1H, m), 3.68 (1H, dd, *J* =10.5, 2.3 Hz), 3.18 (2H, br s), 2.34 (1H, m), 2.13 (1H, m), 1.80 (1H, m), 1.62–1.34 (5H, m), 1.22 (1H, m), 1.17 (3H, d, *J* ***=*** 6.2 Hz), 1.09 (3H, s); ^13^C-NMR (100 MHz, CDCl_3_) δ ppm: 136.1, 118.7, 72.5, 69.2, 58.8, 47.8, 39.5, 37.1, 30.2, 22.7, 22.5, 22.4; HRESIMS (*m*/*z*) calcd. for C_12_H_23_O_2_ (M+H)^+^ 199.1698, found 199.1713; Anal. Calcd. for C_12_H_22_O_2_: C, 72.68; H, 11.18. Found: C, 72.40; H, 10.99. Compound **11b**: mp 93–95 °C; [α]_D_^25^ −35.0 (*c* 0.20, CHCl_3_); IR (KBr) 3351, 2953 cm^−1^; ^1^H-NMR (400 MHz, CDCl_3_) δ ppm: 5.30 (1H, m), 5.18 (1H, d, *J =* 10.2 Hz), 5.17 (1H, d, *J =* 16.9 Hz), 4.11 (1H, m), 3.76 (1H, dd, *J =* 10.8, 2.1 Hz), 2.48 (1H, m), 2.27 (2H, br s), 2.13 (1H, m), 1.85 (1H, m), 1.67 (2H, m), 1.60 (2H, m), 1.45 (2H, m,), 1.22 (3H, d, *J =* 6.2 Hz), 1.19 (3H, s); ^13^C-NMR (100 MHz, CDCl_3_) δ ppm: 136.2, 118.6, 75.7, 69.5, 59.5, 47.5, 37.8, 36.5, 32.0, 29.5, 23.9, 23.4; HRESIMS (*m*/*z*) calcd. for C_12_H_23_O_2_ (M+H)^+^ 199.1698, found 199.1681; Anal. Calcd. for C_12_H_22_O_2_: C, 72.68; H, 11.18. Found: C, 72.65; H, 10.91.

### 3.10. (1R,2R,3aS,3R)-3-Allyl-1,3a-dimethylhexahydrocyclopenta[c]furan (**12a**)

To a solution of diol **11a** (34.4 mg, 0.174 mmol) in CH_2_Cl_2_ (1.7 mL) were added Et_3_N (105 mg, 1.04 mmol), DMAP (106 mg, 0.868 mmol) and *p*-toluenesulfonyl chloride (132 mg, 0.694 mmol) at r.t. The mixture was stirred for two days at the same temperature. The mixture was diluted with Et_2_O, washed with saturated aqueous NH_4_Cl solution, H_2_O and brine, and then dried. Removal of the solvent gave a residue which was then purified by silica gel column chromatography (hexane/AcOEt = 10:1) to generate tetrahydrofuran **12a** (24.1 mg, 77% yield) as a colorless oil. [α]_D_^26^ +21.5 (*c* 1.58, CHCl_3_); IR (neat) 2953, 2870 cm^−1^; ^1^H-NMR (400 MHz, CDCl_3_) δ ppm: 5.84 (1H, m), 5.09 (1H, m), 5.03 (1H, m), 4.25 (1H, quint., *J* = 6.5 Hz), 3.64 (1H, dd, *J* = 9.0, 4.8 Hz), 2.30–2.15 (2H, m), 2.07 (1H, m), 1.68–1.59 (5H, m), 1.46 (1H, m), 1.16 (3H, d, *J* = 6.5 Hz), 1.07 (3H, s); NOESY correlations (H/H): H-1/H-4; H-3/H-17; H-9/H-17; ^13^C-NMR (100 MHz, CDCl_3_) δ ppm: 136.7, 116.4, 85.0, 74.5, 57.0, 39.1, 35.4, 27.3, 26.5, 22.1, 17.3; HRESIMS (*m*/*z*) calcd. for C_12_H_21_O (M+H)^+^ 181.1592, found 181.1590; Anal. Calcd. for C_12_H_20_O: C, 79.94; H, 11.18. Found: C, 80.11; H, 11.10.

### 3.11. (1R,2R,3aS,3S)-3-Allyl-1,3a-dimethylhexahydrocyclopenta[c]furan (**12b**)

To a solution of diol **11b** (32.4 mg, 0.163 mmol) in CH_2_Cl_2_ (1.6 mL) were added Et_3_N (82.8 mg, 0.817 mmol), DMAP (79.9 mg, 0.654 mmol) and *p*-toluenesulfonyl chloride (93.5 mg, 0.490 mmol) at r.t. The mixture was stirred for 2 days at the same temperature. The mixture was diluted with Et_2_O, washed with saturated aqueous NH_4_Cl solution, H_2_O and brine, and then dried. Removal of the solvent gave a residue which was then purified by silica gel column chromatography (hexane/AcOEt = 5:1) to generate tetrahydrofuran **12b** (27.1 mg, 93% yield) as a colorless oil. [α]_D_^26^ −35.1 (*c* 1.14, CHCl_3_); IR (neat) 2951, 2866 cm^−1^; ^1^H-NMR (400 MHz, CDCl_3_) δ ppm: 5.87 (1H, m), 5.12 (1H, m), 5.04 (1H, m), 3.84 (1H, quint., *J* = 6.4 Hz), 3.32 (1H, dd, *J* = 8.4, 4.9 Hz), 2.36–2.23 (2H, m), 1.97 (1H, m), 1.63–1.59 (6H, m), 1.19 (3H, d, *J* = 6.5 Hz), 1.07 (3H, s); NOESY correlations (H/H): H-1/H-4; H-2/H-3; H-2/H-8; H-3/H-17; H-8/H-17; ^13^C-NMR (100 MHz, CDCl_3_) δ ppm: 136.1, 116.3, 86.9, 75.4, 56.1, 35.1, 34.7, 30.0, 27.7, 26.8, 25.9, 15.5; HRESIMS (*m*/*z*) calcd. for C_12_H_21_O (M+H)^+^ 181.1592, found 181.1577; Anal. Calcd. for C_12_H_20_O: C, 79.94; H, 11.18. Found: C, 79.91; H, 11.14.

### 3.12. Conversion from diol **11b** to diol **11a**

To a solution of diol **11b** (50.0 mg, 0.252 mmol) in DMF (252 μL) were added imidazole (21.0 mg, 0.308 mmol) and TBDPSCl (83.5 mg, 0.304 mmol) and the mixture was stirred at r.t. for 1 hr. The mixture was diluted with Et_2_O, washed with saturated aqueous NaHCO_3_ solution, H_2_O and brine and then dried. The crude mixture was diluted with Et_2_O and filtered through a silica gel pad. The filtrate was concentrated to afford the crude alcohol. To a solution of the crude alcohol in CH_3_CN (2.5 mL) was added IBX (212 mg, 0.757 mmol) at r.t. After stirring for 30 min at 80 °C, the mixture was diluted with Et_2_O and then filtered through a Celite pad. Removal of the solvent gave a residue which was filtered through a silica gel pad to afford the crude ketone. To a solution of the crude ketone in MeOH (2.5 mL) was added NaBH_4_ (28.6 mg, 0.756 mmol) at r.t. After stirring for 2 hr under reflux, the mixture was diluted with Et_2_O, washed with H_2_O and brine and then dried. Removal of the solvent gave a residue which was then filtered through a silica gel pad to afford the crude alcohols. To a solution of the crude alcohols in THF (2.5 mL) was added TBAF (760 μL, 0.760 mmol, 1.0 M in THF solution) at r.t. After stirring for 12 hr at 40 °C, the mixture was diluted with Et_2_O, washed with H_2_O and brine and then dried. Removal of the solvent gave a residue which was then purified by silica gel column chromatography (hexane/AcOEt = 4:1) to generate diol **11a** (34.6 mg, 69%) and diol **11b** (6.9 mg, 14 %).

### 3.13. (1S,2R)-1-[(R)-1-(tert-Butyldimethylsiloxy)but-3-enyl]-2-[(S)-1-(tert-butyldimethylsiloxy)ethyl]-1-methylcyclopentane (**13**)

To a solution of diol **11a** (988 mg, 4.98 mmol) in CH_2_Cl_2_ (2.7 mL) were added 2,6-lutidine (2.67 g, 24.9 mmol) and TBSOTf (3.95 g, 14.9 mmol) and the mixture was stirred at 0 °C for 30 min. The mixture was diluted with Et_2_O, washed with saturated aqueous NaHCO_3_ solution, H_2_O and brine, and then dried. Removal of the solvent gave a residue which was then purified by silica gel column chromatography (hexane only) to generate bis-silyl ether **13** (2.11 g, 99% yield) as a colorless oil. [α]_D_^25^ +0.97 (*c* 1.07, CHCl_3_); IR (neat) 2956, 2885, 1471 cm^−1^; ^1^H-NMR (400 MHz, CDCl_3_) δ ppm: 5.90 (1H, m), 5.00 (2H, m), 4.13 (1H, quint, *J* = 6.1 Hz), 3.80 (1H, dd, *J* = 6.5, 3.7 Hz), 2.38 (1H, m), 2.22 (1H, m), 1.85–1.57 (6H, m), 1.19 (1H, m), 1.09 (3H, d, *J* = 6.1 Hz), 1.04 (3H, s), 0.89 (9H, s), 0.88 (9H, s), 0.06 (12H, m); ^13^C-NMR (100 MHz, CDCl_3_) δ ppm: 137.7, 115.8, 77.2, 76.8, 69.1, 56.6, 50.1, 40.3, 35.1, 27.4, 27.1, 26.2, 26.0, 22.5, 18.4, 18.1, −3.0, −3.4, −3.6, −4.0; HRESIMS (*m*/*z*) calcd. for C_24_H_51_O_2_Si_2_ (M+H)^+^ 427.3428, found 427.3437; Anal. Calcd. for C_24_H_50_O_2_Si_2_: C, 67.54; H, 11.81. Found: C, 67.44; H, 11.66.

### 3.14. (R)-3-(tert-Butyldimethylsiloxy)-3-{(1S,2R)-2-[(S)-1-(tert-butyldimethylsiloxy)ethyl]-1-methyl-cyclopentyl}propionaldehyde (**14**)

A cold (−78 °C) solution of bis-silyl ether **13** (482 mg, 1.13 mmol) in CH_2_Cl_2_ (56.5 mL) was treated with ozone until the blue color generated persisted for more than 15 min. Excess ozone was removed using an argon flow. To the mixture were then added MeOH (56.5 mL), Zn powder (739 mg, 11.3 mmol), KI (1.88 g, 11.3 mmol) and AcOH (682 mg, 11.4 mmol). The mixture was allowed to warm to r.t., stirred for 1 hr at the same temperature and then concentrated under reduced pressure. The resultant residue was diluted with Et_2_O, washed with saturated aqueous NaHCO_3_ solution, H_2_O and brine and then dried. Removal of the solvent gave a residue which was then purified by silica gel column chromatography (hexane/AcOEt = 20:1) to generate aldehyde **14** (484 mg, quantitative yield) as a colorless oil. [α]_D_^25^ −0.56 (*c* 1.08, CHCl_3_); IR (neat) 2955, 2857, 1727 cm^−1^; ^1^H-NMR (400 MHz, CDCl_3_) δ ppm: 9.85 (1H, dd, *J* = 2.7, 1.3 Hz), 4.42 (1H, dd, *J =* 6.0, 4.1 Hz), 4.10 (1H, quint, *J =* 6.1 Hz), 2.67 (2H, m), 1.84 (1H, m), 1.74 (2H, m), 1.61 (3H, m), 1.23 (1H, m), 1.12 (3H, d, *J* = 6.1 Hz), 1.05 (3H, s), 0.88 (9H, s), 0.88 (9H, s), 0.06 (12H, m); ^13^C-NMR (100 MHz, CDCl_3_) δ ppm: 201.9, 70.9, 69.0, 56.3, 50.5, 49.6, 35.4, 27.3, 26.7, 26.0, 22.4, 22.3, −3.4, −3.7, −4.0, −4.1; HRESIMS (*m*/*z*) calcd. for C_23_H_48_O_3_Si_2_Na (M+Na)^+^ 451.3040, found 451.3052; Anal. Calcd. for C_23_H_48_O_3_Si_2_: C, 64.42; H, 11.28. Found: C, 64.40; H, 11.10.

### 3.15. (6E,9R)-9-(tert-Butyldimethylsiloxy)-9-{(1R,2S)-2-[(S)-1-(tert-butyldimethylsiloxy)ethyl]-1-methylcyclopentyl}-2,6-dimethylnona-2,6-dien-5-one (**16**)

To a solution of phosphonate **15** [5] (306 mg, 1.17 mmol) in THF (700 μL) was added *^n^*BuLi (591 μL, 0.935 mmol, 1.58 M in hexane solution) at 0 °C. The mixture was stirred for 1 hr at the same temperature and a solution of aldehyde **14** (200 mg, 0.467 mmol) in THF (4.0 mL) was added dropwise at r.t. After stirring for 5 hr, the mixture was diluted with Et_2_O, washed with saturated aqueous NH_4_Cl solution, H_2_O and brine, and then dried. Removal of the solvent gave a residue which was then purified by silica gel column chromatography (hexane/benzene = 4:1) to generate α,β-unsaturated ketone **16** (123 mg, 49% yield) as a white solid and recovered aldehyde **14** (36.5 mg, 18% yield). Compound **16**: mp 55–58 °C; [α]_D_^25^ +11.2 (*c* 0.57, CHCl_3_); UV (MeOH) λ_max_ (ɛ) nm: 234 (18800); IR (KBr) 2956, 2931, 2856, 1674 cm^−1^; ^1^H-NMR (400 MHz, CDCl_3_) δ ppm: 6.83 (1H, t, *J =* 6.1 Hz), 5.34 (1H, m), 4.10 (1H, quint, *J =* 6.2 Hz), 3.97 (1H, t, *J =* 5.6 Hz), 3.38 (2H, d, *J =* 7.0 Hz), 2.49 (2H, m), 1.85 (1H, m), 1.78 (3H, s), 1.74 (3H, s), 1.80–1.74 (2H, m), 1.64 (3H, s), 1.64–1.57 (4H, m), 1.11 (3H, d, *J =* 6.1 Hz), 1.06 (3H, s), 0.90 (9H, s), 0.88 (9H, s), 0.08 (6H, d, *J =* 12.6 Hz), 0.06 (6H, d, *J =* 6.5 Hz); ^13^C-NMR (100 MHz, CDCl_3_) δ ppm: 199.9, 141.5, 136.9, 134.6, 117.1, 77.2, 75.9, 69.1, 56.5, 50.1, 37.1, 35.6, 35.1, 27.2, 27.1, 26.1, 26.0, 25.7, 22.5, 22.4, 18.3, 18.1, 11.9, −3.2, −3.4, −3.8, −3.9; HRESIMS (*m*/*z*) calcd. for C_31_H_61_O_3_Si_2_ (M+H)^+^ 537.4159, found 537.4168; Anal. Calcd. for C_31_H_60_O_3_Si_2_: C, 69.34; H, 11.26. Found: C, 69.40; H, 11.05.

### 3.16. (5R,6E,9R)-9-(tert-Butyldimethylsiloxy)-9-{(1R,2S)-2-[(S)-1-(tert-butyldimethylsiloxy)ethyl]-1-methylcyclopentyl}-2,6-dimethylnona-2,6-dien-5-ol and (5S,6E,9R)-9-(tert-butyldimethylsiloxy)-9-{(1R,2S)-2-[(S)-1-(tert-butyldimethylsiloxy)ethyl]-1-methylcyclopentyl}-2,6-dimethylnona-2,6-dien-5-ol (**17**)

To a solution of CeCl_3_·7H_2_O (144 mg, 0.386 mmol) in MeOH (9.3 mL) was added NaBH_4_ (11.0 mg, 0.290 mmol) at 0 °C. The mixture was then added to a solution of α,β-unsaturated ketone **16** (104 mg, 0.193 mmol) in MeOH (10.0 mL) at 0 °C and stirred for 30 min at the same temperature. The mixture was diluted with Et_2_O, washed with H_2_O and brine, and then dried. Removal of the solvent gave a residue which was then purified by silica gel column chromatography (CHCl_3_ only) to generate a diastereomeric mixture of allylic alcohol **17** (103 mg, 99% yield) as a colorless oil. IR (neat) 3353, 2957 cm^−1^; ^1^H-NMR (400 MHz, CDCl_3_) δ ppm: 5.54 (1H, m), 5.11 (1H, m), 4.14 (1H, m), 4.00 (1H, m), 3.80 (1H, m), 2.42–2.14 (4H, m), 1.86 (1H, m), 1.80–1.57 (5H, m), 1.72 (3H, s), 1.64 (3H, s), 1.62 (3H, s), 1.15 (1H, m), 1.09 (3H, d, *J* = 6.1 Hz), 1.02 (1.5 H, s), 1.02 (1.5H, s), 0.88 (9H, d, *J* = 6.9 Hz), 0.88 (9H, d, *J* = 5.4 Hz), 0.06 (12H, m); ^13^C-NMR (100 MHz, CDCl_3_) δ ppm: 136.6, 136.5, 134.8, 134.7, 125.6, 125.5, 120.2, 120.2, 77.2, 77.2, 68.9, 68.9, 56.2, 56.2, 50.2, 50.2, 35.1, 35.0, 34.1, 34.0, 26.7, 26.6, 26.5, 26.3, 26.2, 26.0, 25.9, 22.5, 22.1, 22.1, 22.1, 18.4, 18.1, 18.0, 12.1, 12.1, −3.1, −3.2, −3.4, −3.5, −3.7, −3.7, −4.0, −4.0; HRESIMS (*m*/*z*) calcd. for C_31_H_62_O_3_Si_2_Na (M+Na)^+^ 561.4135, found 561.4156; Anal. Calcd. for C_31_H_62_O_3_Si_2_: C, 69.08; H, 11.59. Found: C, 68.92; H, 11.30.

### 3.17. (1R,3E,5R)-1-[(1S,2R)-2-((S)-1-Hydroxyethyl)-1-methylcyclopentyl]-4,8-dimethyl-5-trityloxy-nona-3,7-dien-1-ol and (1R,3E,5S)-1-[(1S,2R)-2-((S)-1-hydroxyethyl)-1-methylcyclopentyl]-4,8-dimethyl-5-trityloxynona-3,7-dien-1-ol (**18**)

To a solution of the diastereomeric mixture of allylic alcohol **17** (40.0 mg, 0.074 mmol) in pyridine (740 μL) were added DMAP (5.0 mg, 0.041 mmol) and TrCl (103 mg, 0.395 mmol) at r.t. After stirring for 4 days at 80 °C, the mixture was diluted with Et_2_O, washed with H_2_O and brine, and then dried. Removal of the solvent gave a residue which was filtered through a short-path silica gel pad (hexane/AcOEt = 20:1). The filtrate was then concentrated to afford the crude trityl ether. To a solution of the crude trityl ether in DMF (1.5 mL) was added TBAF (1.5 mL, 0.150 mmol, 1.0 M in THF solution) at r.t. After stirring for 2 days at 50 °C, the mixture was diluted with Et_2_O, washed with H_2_O and brine and then dried. Removal of the solvent gave a residue which was then purified by silica gel column chromatography (hexane/AcOEt = 10:1) to generate a diastereomeric mixture of diol **18** (40.9 mg, quantitative yield) as a colorless oil. IR (neat) 3344, 2961 cm^−1^; ^1^H-NMR (400 MHz, CDCl_3_) δ ppm: 7.51 (6H, m), 7.30–7.20 (9H, m), 4.93 (1H, t, *J* = 6.6 Hz), 4.77 (0.5H, dd, *J* = 6.7, 6.7 Hz), 4.68 (0.5H, dd, *J* = 9.0, 5.5 Hz), 4.00 (1H, dd, *J* = 5.7, 5.7 Hz), 3.79 (0.5H, m), 3.73 (0.5H, m), 3.47–3.36 (1H, m), 2.31 (0.5H, m), 2.12–1.88 (3.5 H, m), 1.82–1.73 (2.5H, m), 1.63 (3H, m), 1.54 (3H, m), 1.48 (3H, m), 1.57–1.43 (2.5H, m), 1.41–1.31 (2H, m), 1.17 (1.5H, m), 1.14 (1.5H, m), 1.07 (3H, m); ^13^C-NMR (100 MHz, CDCl_3_) δ ppm: 145.1, 140.4, 140.3, 133.7, 133.2, 129.0, 127.5, 127.5, 126.9, 126.9, 122.1, 121.4, 120.6, 120.4, 87.2, 87.2, 79.5, 78.5, 77.2, 72.6, 72.3, 69.0, 68.9, 59.1, 47.3, 47.2, 39.6, 33.1, 33.0, 30.3, 30.1, 30.1, 29.6, 29.2, 26.1, 25.8, 25.7, 22.4, 22.4, 22.3, 22.3, 22.2, 17.7, 12.7, 11.6; HRESIMS (*m*/*z*) calcd. for C_38_H_48_O_3_Na (M+Na)^+^ 575.3501, found 575.3522; Anal. Calcd. for C_38_H_48_O_3_: C, 82.56; H, 8.75. Found: C, 82.52; H, 8.60.

### 3.18. (3E,5R)-1-((1S,2R)-2-Acetyl-1-methylcyclopentyl)-4,8-dimethyl-5-trityloxynona-3,7-dien-1-one and (3E,5S)-1-((1S,2R)-2-acetyl-1-methylcyclopentyl)-4,8-dimethyl-5-trityloxynona-3,7-dien-1-one (**19**)

To a cold (−78 °C) solution of TFAA (67.6 mg, 0.322 mmol) in CH_2_Cl_2_ (100 μL) was added DMSO (33.6 mg, 0.430 mmol) in CH_2_Cl_2_ (100 μL). The mixture was stirred at 78 °C for 30 min, treated with a solution of the diastereomeric mixture of diol **18** (29.6 mg, 0.054 mmol) in CH_2_Cl_2_ (340 μL), stirred for 2 hr and then Et_3_N (54.3 mg, 0.537 mmol) was added. The mixture was warmed to r.t. and stirred for 30 min. The mixture was diluted with Et_2_O, washed with saturated aqueous NaHCO_3_ solution, H_2_O and brine and then dried. Removal of the solvent gave a residue which was then purified by silica gel column chromatography (hexane/AcOEt = 6:1) to generate a diastereomeric mixture of diketone **19** (27.9 mg, 95% yield) as a colorless oil. IR (neat) 2965, 1705 cm^−1^; ^1^H-NMR (400 MHz, CDCl_3_) δ ppm: 7.49 (6H, m), 7.26–7.16 (9H, m), 4.95 (1H, t, *J* = 6.1 Hz), 4.81 (1H, t, *J* = 7.3 Hz), 3.92 (1H, dd, *J* = 8.8, 4.7 Hz), 2.92 (2H, m), 2.78 (1H, dd, *J* = 8.6, 5.0 Hz), 2.23–1.72 (6H, m), 2.15 (3H, s), 1.63–1.55 (2H, m), 1.58 (3H, s), 1.48 (3H, s), 1.42 (3H, s), 1.21 (3H, s); ^13^C-NMR (100 MHz, CDCl_3_) δ ppm: 212.2, 210.7, 145.2, 137.6, 132.4, 129.2, 127.5, 126.8, 120.5, 118.7, 87.2, 79.3, 77.2, 60.9, 59.8, 37.9, 35.4, 33.5, 29.9, 27.6, 25.8, 25.2, 22.4, 17.8, 12.1; HRESIMS (*m*/*z*) calcd. for C_38_H_44_O_3_Na (M+Na)^+^ 571.3188, found 571.3196. Anal. Calcd. for C_38_H_44_O_3_: C, 83.17; H, 8.08. Found: C, 83.12; H, 8.21.

### 3.19. Diastereomeric mixture of secoxestenone (**20**)

To a solution of the diastereomeric mixture of diketone **19** (76.2 mg, 0.139 mmol) in CH_2_Cl_2_ (14 mL) was added Yb(OTf)_3_ (172 mg, 0.277 mmol) at r.t. After stirring for 30 min, the mixture was diluted with Et_2_O. To this was added NaHCO_3_ and the mixture was then filtered through a silica gel pad. Removal of the solvent gave a residue which was then purified by silica gel column chromatography (hexane/AcOEt = 1:1) to generate a diastereomeric mixture of secoxestenone **20** (33.5 mg, 79% yield) as a colorless oil. IR (neat) 3448, 2965, 1706 cm^−1^; ^1^H-NMR (400 MHz, CDCl_3_) δ ppm: 5.60 (1H, m), 5.10 (1H, m), 4.05 (1H, m), 3.27 (2H, m), 2.85 (1H, m), 2.27 (3H, m), 2.16 (3H, m), 2.09 (1H, m), 1.91–1.75 (3H, m), 1.71 (3H, s), 1.66 (1H, m), 1.63 (3H, s), 1.63 (3H, s), 1.28 (3H m); ^13^C-NMR (100 MHz, CDCl_3_) δ ppm: 212.8, 210.8, 148.5, 141.9, 134.7, 120.8, 120.1, 118.2, 77.2, 76.9, 61.3, 60.4, 59.7, 37.8, 35.9, 35.5, 34.0, 30.2, 29.8, 27.8, 27.7, 25.9, 25.7, 25.6, 25.3, 22.4, 18.0, 12.3, 12.1; HRESIMS (*m*/*z*) calcd. for C_19_H_30_O_3_Na (M+Na)^+^ 329.2093, found 329.2085. Anal. Calcd. for C_19_H_30_O_3_: C, 74.47; H, 9.87. Found: C, 74.29; H, 9.85.

### 3.20. Xestenone (**21**) and 12-epi-xestenone (12-epi-**21**)

To a solution of the diastereomeric mixture of secoxestenone **20** (26.5 mg, 0.087 mmol) in MeOH (6.7 mL) was added 0.1 M NaOH aqueous solution (21.6 mL) at r.t. The mixture was stirred for 30 min, neutralized with 1.0 M HCl aqueous solution, diluted with Et_2_O, washed with H_2_O and brine, dried and then concentrated under reduced pressure. The resultant residue was purified by silica gel column chromatography (hexane/AcOEt = 4:1) to give a mixture of **21** and 12-*epi*-**21** (22.0 mg, 88% yield) as a colorless oil. The above mixture was subjected to HPLC (CHIRALPAK IA, 1.0 cm × 25 cm, hexane/EtOH = 95:5, flow rate: 1.0 mL/min) to give xestenone **21** (*t*_R_ = 12.0 min) and 12-*epi*-**21** (*t*_R_ = 15.0 min); **21**: [α]_D_^25^ +2.2 (*c* 0.075, MeOH); UV (sh, MeOH) λ_max_ nm (ɛ): 257 (6100); CD (MeOH) λ_ext_ nm [θ]: 323 (+87,000), 258 (−129,000); IR (neat) 3419, 1685 cm^−1^; ^1^H-NMR (600 MHz, CDCl_3_) δ ppm: 5.93 (1H, s), 5.18 (1H, br t, *J* = 6.9 Hz), 4.18 (1H, t, *J* = 6.4 Hz), 2.71 (1H, d, *J* = 9.1 Hz), 2.35 (2H, m), 1.96 (3H, s), 1.93 (1H, m), 1.90 (1H, br s), 1.82 (1H, m), 1.75 (3H, s), 1.69 (1H, m), 1.67 (3H, s), 1.64 (1H, m), 1.55 (3H, s), 1.35 (1H, m), 1.25 (1H, m), 1.21 (3H, s); ^13^C-NMR (150 MHz, CDCl_3_) δ ppm: 212.7, 172.2, 144.3, 137.4, 134.9, 119.9, 115.6, 76.4, 56.7, 54.8, 37.5, 34.2, 28.9, 25.9, 24.8, 22.5, 18.0, 16.7, 14.4; HRESIMS (*m*/*z*) calcd. for C_19_H_28_O_2_Na (M+Na)^+^ 311.1987, found 311.1981. Anal. Calcd. for C_19_H_28_O_2_: C, 79.12; H, 9.78. Found: C, 78.97; H, 9.74. 12-*epi*-**21**: [α]_D_^25^ −113.7 (*c* 0.085, MeOH); UV (sh, MeOH) λ_max_ nm (ɛ): 254 (2,100); CD (MeOH) λ_ext_ nm [θ]: 320 (+116,000), 256 (−104,000); IR (neat) 3418, 1686 cm^−1^; ^1^H-NMR (600 MHz, CDCl_3_) δ ppm: 5.92 (1H, s), 5.16 (1H, t, *J* = 7.2 Hz), 4.19 (1H, t, *J* = 6.3 Hz), 2.71 (1H, d, *J* = 9.1 Hz), 2.35 (2H, m), 1.96 (3H, s), 1.93 (1H, m), 1.81 (1H, m), 1.74 (3H, s), 1.69 (1H, m), 1.67 (3H, s), 1.62 (1H, m), 1.55 (3H, s), 1.35 (1H, m), 1.25 (1H, m), 1.22 (3H, s); ^13^C-NMR (150 MHz, CDCl_3_) δ ppm: 212.8, 172.2, 144.3, 137.4, 134.8, 119.9, 115.9, 76.5, 56.7, 54.7, 37.5, 34.1, 28.9, 25.9, 24.8, 22.5, 18.0, 16.7, 14.1; HRESIMS (*m*/*z*) calcd. for C_19_H_28_O_2_Na (M+Na)^+^ 311.1987, found 311.1975. Anal. Calcd. for C_19_H_28_O_2_: C, 79.12; H, 9.78. Found: C, 79.03; H, 9.88.

### 3.21. General procedure for the synthesis of MPA ester

To a solution of xestenone (**21**) in CH_2_Cl_2_ were added DCC, DMAP and (*S*)-(+)- or (*R*)-(−)-α-methoxyphenylacetic acid at r.t. After stirring for 30 min at 40 °C the mixture was concentrated. Removal of the solvent gave a residue which was then purified by silica gel column chromatography (hexane/AcOEt = 6:1) to generate (*S*)- or (*R*)-MPA ester. (*S*)-MPA ester: ^1^H-NMR (400 MHz, CDCl_3_) δ ppm: 7.44–7.27 (5H, m), 5.66 (1H, s), 5.26 (1H, dd, *J* = 7.9, 5.7 Hz), 5.06 (1H, m), 4.75 (1H, s), 3.43 (3H, s), 2.62 (1H, d, *J* = 9.2 Hz), 2.39 (2H, m), 1.88 (1H, m), 1.76 (1H, m), 1.70 (3H, s), 1.68 (3H, s), 1.61 (3H, s), 1.59 (2H, m), 1.30 (1H, m), 1.26 (3H, br s), 1.19 (1H, m), 1.15 (3H, s); ^13^C-NMR (150 MHz, CDCl_3_) δ ppm: 211.9, 169.8, 139.2, 137.0, 134.6, 128.8, 128.5, 128.2, 127.2, 119.0, 118.1, 117.8, 82.6, 79.0, 56.6, 54.7, 37.4, 31.8, 29.7, 28.8, 25.8, 24.7, 22.5, 18.0, 16.5, 14.4; HRESIMS (*m*/*z*) calcd. for C_28_H_36_O_4_Na (M+Na)^+^ 459.2511, found 459.2521; (*R*)-MPA ester: ^1^H-NMR (400 MHz, CDCl_3_) δ ppm: 7.45–7.29 (5H, m), 5.88 (1H, s), 5.24 (1H, dd, *J* = 7.7, 5.7 Hz), 4.80 (1H, m), 4.77 (1H, s), 3.43 (3H, s), 2.67 (1H, d, *J* = 9.0 Hz), 2.28 (2H, m), 1.91 (1H, m), 1.85 (3H, s), 1.81–1.59 (3H, m), 1.53 (3H, s), 1.48 (3H, br s), 1.47 (3H, s), 1.33 (1H, m), 1.22 (1H, m), 1.19 (3H, s); ^13^C-NMR (150 MHz, CDCl_3_) δ ppm: 212.1, 170.0, 139.5, 137.0, 134.4, 128.8, 128.5, 128.2, 127.2, 119.0, 118.7, 117.8, 82.6, 79.0, 56.7, 54.7, 37.5, 31.9, 29.7, 28.9, 25.6, 24.8, 22.5, 17.8, 16.6, 16.5; HRESIMS (*m*/*z*) calcd. for C_28_H_36_O_4_Na (M+Na)+ 459.2511, found 459.2501.

### 3.22. Conversion of 12-epi-**21** to **21**

To a solution of 12-*epi*-**21** (1.7 mg, 0.006 mmol) in THF (59 μL) were added Ph_3_P (2.3 mg, 0.009 mmol) and *p*-NO_2_BzOH (1.5 mg, 0.009 mmol) at r.t. After stirring for 10 min, DIAD (1.8 mg, 0.009 mmol) was added and the mixture was stirred for an additional 2 hr. The crude mixture was diluted with Et_2_O and filtered through a silica gel pad. The filtrate was then concentrated to afford the crude ester. To a solution of the crude ester in MeOH (200 μL) was added K_2_CO_3_ (12.2 mg, 0.088 mmol) at r.t. After stirring for 30 min at the same temperature, the mixture was diluted with Et_2_O and then filtered through a silica gel pad. Removal of the solvent gave a residue which was then purified by silica gel column chromatography (hexane/AcOEt = 2:1) to generate xestenone (**21**, 1.6 mg, 95% yield).

## 4. Conclusions

The first total synthesis of xestenone has been accomplished *via* the stereocontrolled one-pot synthesis of cyclopentane derivatives using allyl phenyl sulfone as the key step. Moreover, the authors have determined that the absolute configuration of xestenone is 3*S*, 7*S* and 12*R*.
